# Retroiridian Pupilloplasty for Phakic Traumatic Pseudoacorea: A Novel Minimally Invasive Surgical Technique

**Published:** 2025-07-22

**Authors:** Andres German Alza

**Affiliations:** Private Eye Clinic Dr. Enrique Alza, La Plata, Buenos Aires, Argentina

**Keywords:** Pseudoacorea, Ocular trauma, Healthy lens, Phakic retroiridian pupilloplasty

## Abstract

We describe a novel, minimally invasive surgical technique termed retroiridian pupilloplasty, performed in a phakic patient with a traumatic pseudoacorea of 58 years’ duration. This reproducible approach, first published of us in 2022, uses an anterior vitrector to create a neopupil without a preexisting pupillary opening through the retroiridian chamber. The procedure significantly improved visual function and cosmesis, with minimal postoperative care and rapid recovery. In this case, the procedure resulted in significant improvements in both visual function and cosmetic appearance, required minimal postoperative follow-up, and allowed for rapid recovery. From the surgeon’s perspective, it was a straightforward, effective, and safe approach, representing a simple and viable therapeutic option for similar cases.

## INTRODUCTION

Pseudoacoria, or “hidden pupil,” is a rare condition in which the pupil is not visible at rest and only becomes apparent after pharmacologic mydriasis. This distinguishes it from true acoria, where the pupil is completely absent. We present the case of a patient with traumatic pseudoacoria and an intact crystalline lens who was treated using an innovative surgical technique: *Phakic retroiridian pupilloplasty*. Unlike conventional pupilloplasty techniques, this approach is performed through the retroiridian space. Its effectiveness lies in its ability to restore the anatomical structure of the pupil while providing a practical and straightforward solution for this type of case. As the second case in our surgical series, it underscores both the feasibility and therapeutic potential of phakic retroiridian pupilloplasty for the treatment of pseudoacoria.

## CASE REPORT

**Figure 1:**
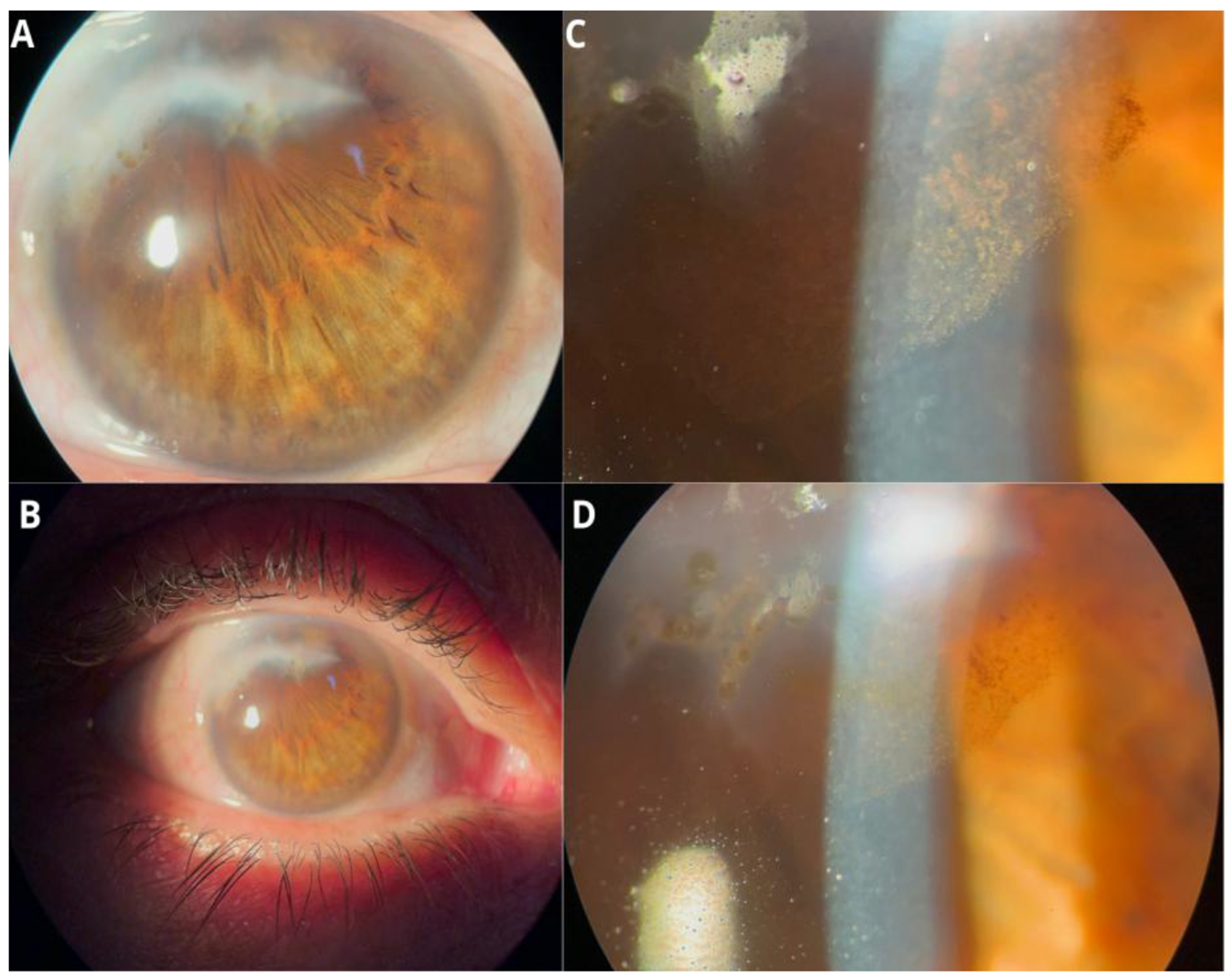
**(A-D):** Slit-lamp biomicroscopy revealed pseudoacorea with an extensive superior corneal scar and endothelial involvement.

**Figure 2:**
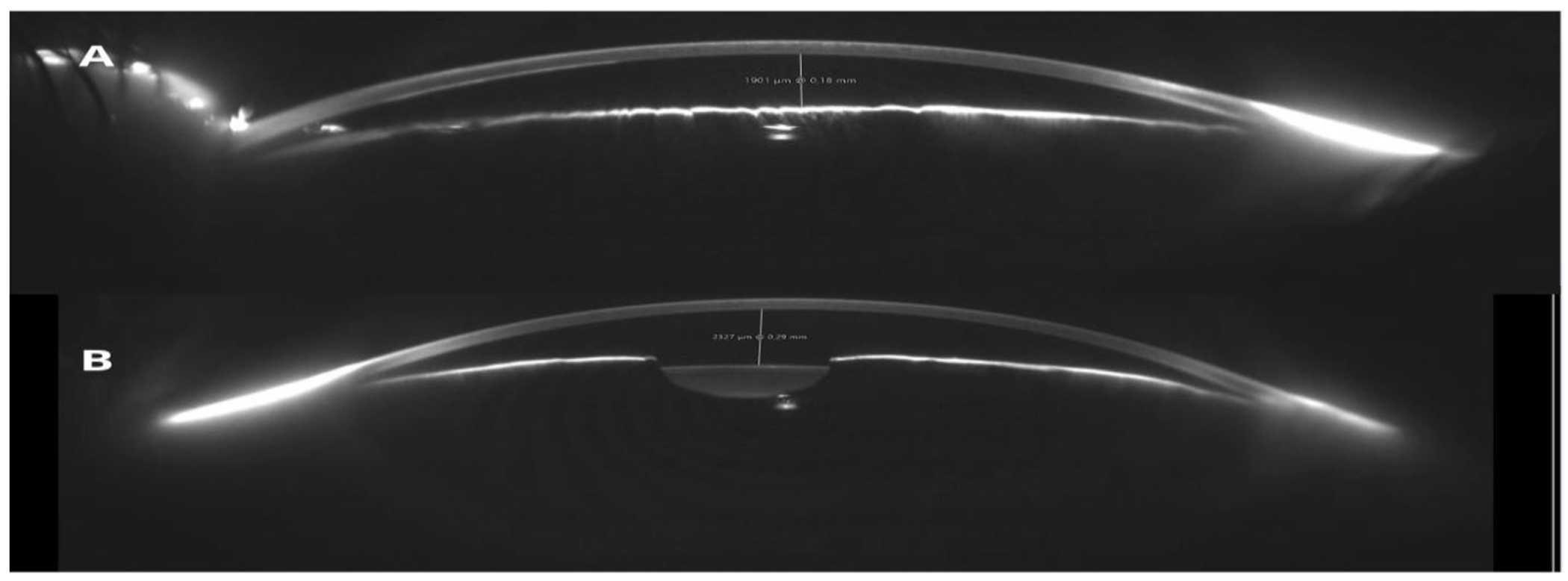
**(A)** Anterior segment evaluation using Scheimpflug imaging revealed the absence of a visible pupil. **(B)** Following retroiridian pupilloplasty, a neopupil was observed.

**Figure 3:**
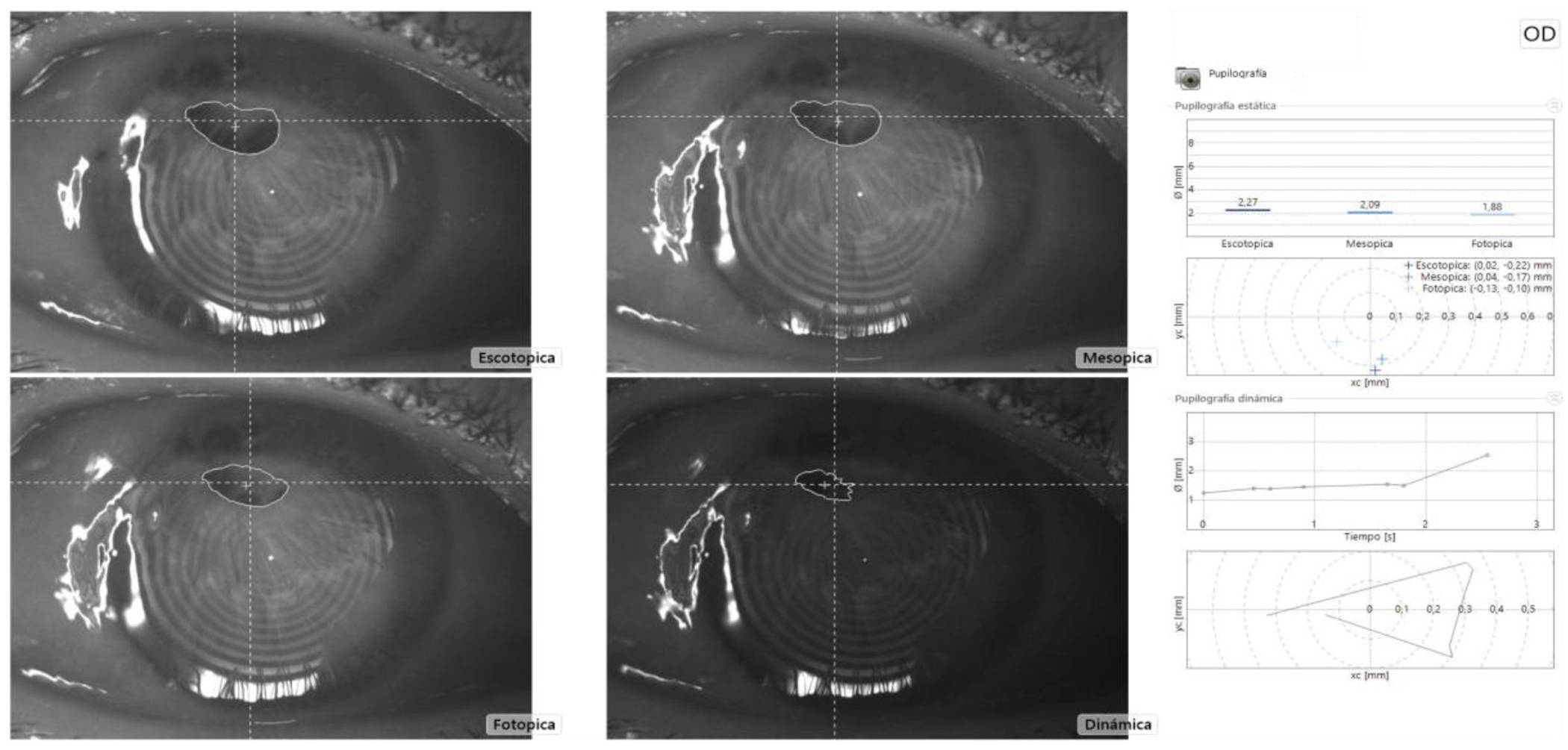
Measurement of the pupillary response in various lighting conditions, prior to pupilloplasty.

**Figure 4:**
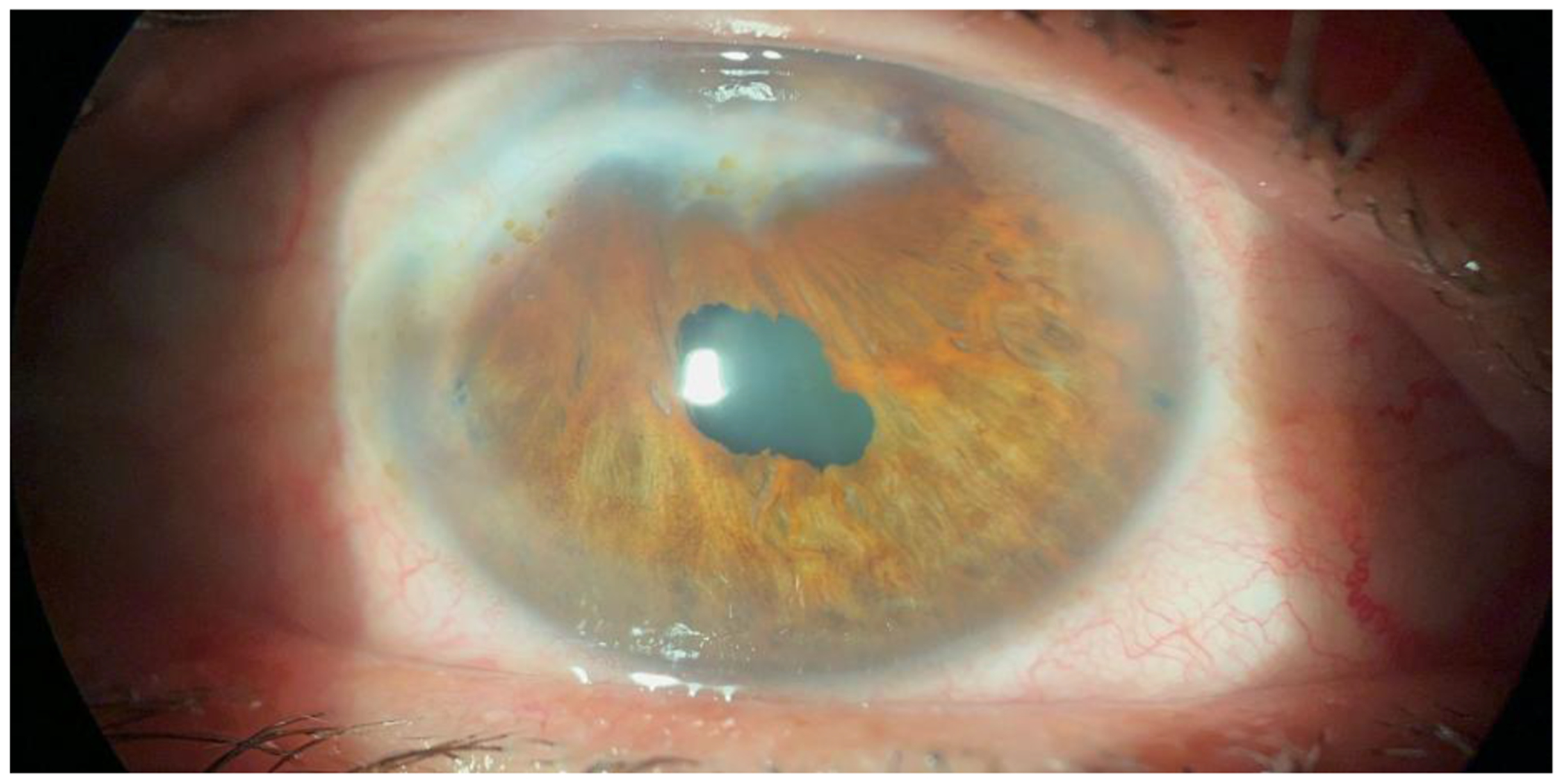
Slit-lamp photographs following phakic retroiridian pupilloplasty.

We present the case of a 62-year-old male patient with acquired pseudoacoria in the right eye, resulting from blunt ocular trauma caused by a stone impact 58 years prior. Initially, visual acuity was limited to light perception. Slit-lamp bio microscopy revealed pseudoacoria with an extensive superior corneal scar and endothelial involvement (Figure 1A – Figure 1D). Evaluation with a Scheimpflug camera confirmed the apparent absence of a visible pupil (Figure 2A). This equipment was also useful for assessing pupillary location and reactivity through the corneal opacity. This allowed for the identification of a small, corectopic pupil displaced superiorly (Figure 3). Based on these findings, we selected phakic retroiridian pupilloplasty as the surgical approach (Figure 4). Postoperatively, the patient experienced a significant improvement in visual acuity, achieving a LogMAR of 0.22. Follow-up imaging with Scheimpflug camera (Figure 2B) confirmed the procedure’s success.

A functional pupil with a sufficient diameter for refraction was obtained along with excellent residual visual acuity. These findings underscore the effectiveness of this technique in restoring visual function in cases of traumatic pseudoacoria with a preserved crystalline lens.

### Surgical Technique

**Figure 5:**
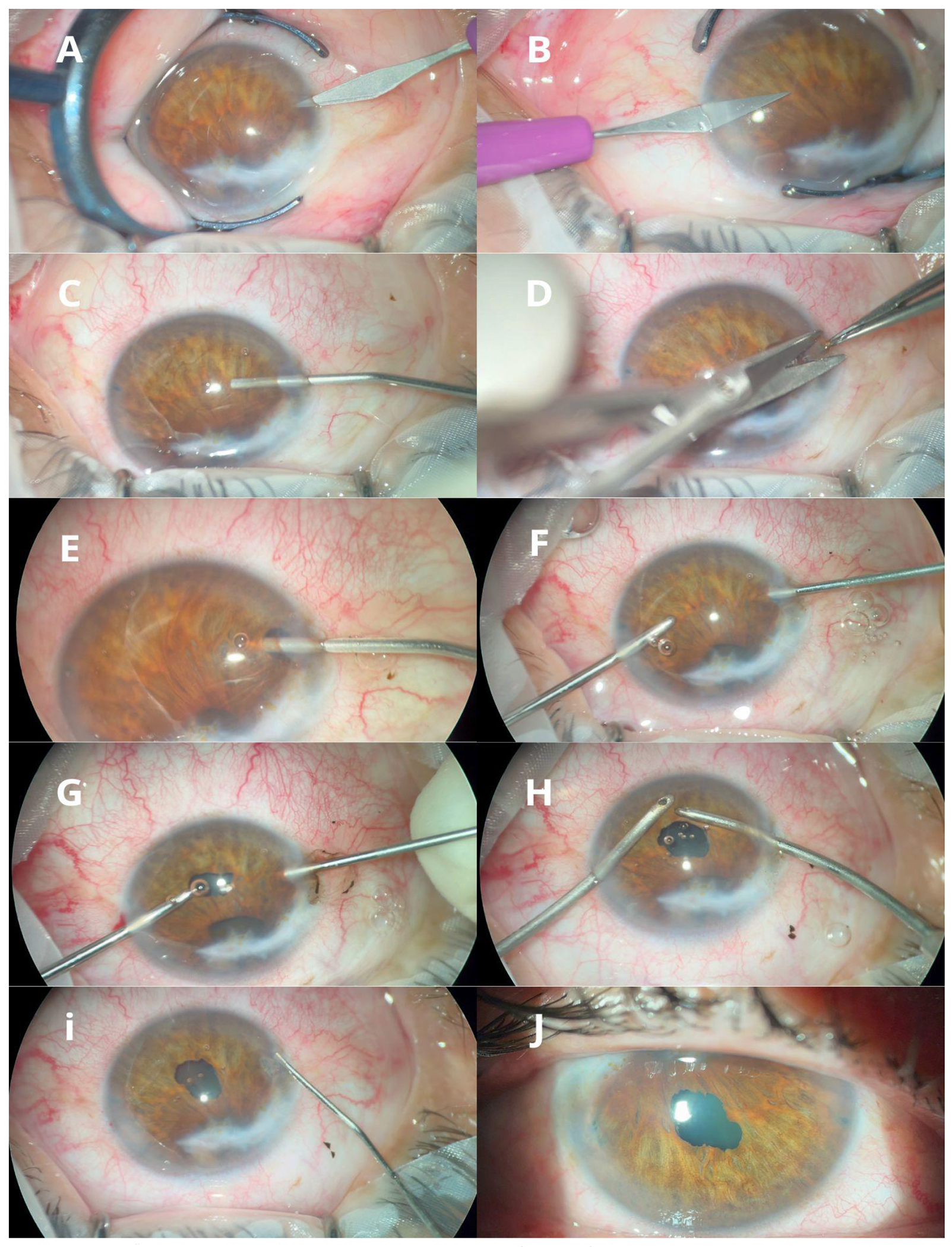
(**A-J)**. Sequential images illustrating the step-by-step technique of phakic retroiridian pupilloplasty. Two 0.8 mm sclero-corneal incisions were made at the temporal and nasal positions using a 15° blade (**A-B**). One-third of the anterior chamber was filled with 2% hydroxypropyl methylcellulose to protect the corneal endothelium (**C**). A peripheral iridectomy was performed using Westcott scissors and Castroviejo forceps to gain access to the retroiridian space (**D**). This space was then expanded with 3% sodium hyaluronate, forming a convex dome occupying two-thirds of the anterior chamber without contacting the endothelium (**E**). A 20-gauge anterior chamber vitrectome was introduced through the iridectomy; its silhouette was visualized through the iris to ensure avoidance of contact with the crystalline lens (**F**). Phakic retroiridian pupilloplasty was then performed (**G**). The viscoelastic material was aspirated from the anterior chamber (**H**). The incisions were hydrated, leaving the iris engaged at the site of the iridectomy (**I**). The final image shows the resulting neopupil (**J**).

The procedure began with local antisepsis and topical anesthesia. Two 0.8 mm sclero-corneal incisions were made (temporal and nasal), and one-third of the anterior chamber was filled with 2% hydroxypropyl methylcellulose to protect the endothelium. A peripheral iridectomy was then performed using Westcott scissors and Castroviejo forceps to access the retroiridian space. This retroiridian space was expanded by injecting 3% sodium hyaluronate, creating a dome that occupied two-thirds of the anterior chamber without contacting the endothelium. A 20G anterior vitrectome was introduced through the iridectomy and angled upward so that its silhouette was visible through the iris, avoiding contact with the crystalline lens. The phakic retroiridian pupilloplasty was performed using the following parameters: a cutting rate of 1000 cpm, aspiration rate of 20 cc/min, and vacuum pressure of 250 mmHg. At the end of the procedure, the viscoelastic was aspirated, the incisions were hydrated, and the iris was left engaged at the iridectomy site, eliminating the need for sutures. This resulted in the successful creation of a neopupil (Table 1, Figure 5A – Figure 5J).

### Postoperative

The patient received topical treatment with combined ciprofloxacin 0.3% and dexamethasone 0.1% eye drops for 16 days. The initial dosage was four times daily for the first four days, reducing to twice daily for the remaining twelve days. Follow-up visits were scheduled at 24 hours, 5 days, and 1 month.

## DISCUSSION

The correction of pupillary abnormalities, regardless of their etiology, presents a significant surgical challenge, particularly in cases of pseudoacoria or acoria. Various approaches have traditionally been employed (Table 2), including Nd:YAG laser disruption [1], as well as the use of a vitrectome, microforceps, and microsurgical scissors [2,3]. Among the techniques described, Kandori et al. [4] proposed the use of a 25G vitrector for membrane removal. However, caution was taken to avoid damaging the anterior surface of the lens; therefore, the white membrane adherent to the lens was left in place [4]. Another option is iris microendodiathermy or endothermal pupilloplasty [5], often combined with Siepser sutures [6] to reshape the pupil through tissue contraction induced by a bipolar cautery device. However, this latter method generates high temperatures, posing risks of damage to adjacent tissues, long-term iris depigmentation, and limited efficacy in cases of acoria. A common limitation shared by all these techniques is their anterior chamber approach, which increases the risk of accidental contact with the anterior lens capsule [7,8] and reduces intraoperative visibility. In this context, phakic retroiridian pupilloplasty emerges as an innovative surgical technique. Its goal is to create a new pupil in patients with pseudoacoria or a “hidden pupil,” while preserving the natural crystalline lens. Unlike conventional pupilloplasty techniques, this approach is performed through the retroiridian space [9], which offers a natural dissection plane, requires no additional instrumentation, and provides a distinct and potentially safer access route (Table 3). Our 2022 report was, to our knowledge, the first to describe a reproducible, minimally invasive retroiridian approach using an anterior vitrector in a phakic eye without preexisting pupillary opening. The technique included detailed steps, safety measures, and postoperative outcomes with preserved lens clarity and improved cosmesis and was initially applied in a patient with pseudoacoria secondary to Axenfeld-Rieger syndrome [10,11]. Specifically, Bobrova et al. [12] describe an adaptation that used our approach of working through the retroiridian space in a phakic eye, including the entry gate and dome formation. The variation involved the use of a spatula, a vitrectome, and microsurgical scissors within the anterior chamber. Essentially, the main difference lies in the use of additional instruments to treat a specific membrane that was not addressed in the original technique, which caused serious issues such as pupil absence and blockage of aqueous humor drainage. Therefore, this new variation expands the scope and applicability of the original technique.

This second surgical case in our series, along with others employing our technique but with different etiologies than the one originally reported in 2022, further supports the feasibility and potential of phakic retroiridian pupilloplasty as an effective technique for correcting pseudoacoria.

## CONCLUSION

To our knowledge, the first description of a reproducible and minimally invasive retroiridian approach using an anterior vitrector in a phakic eye without a preexisting pupillary opening was published in our 2022 report. Since then, the technique has proven to be safe, consistent, and effective in cases of both congenital and acquired pseudoacorea. Phakic retroiridian pupilloplasty is an innovative, effective, and safe surgical technique for the creation of a neopupil. It has been successfully performed in adult patients with both congenital and acquired pseudoacorea and may also be considered in cases of acorea. With over three years of follow-up, this approach has demonstrated not only significant improvements in visual function and cosmetic outcomes but also promising potential in pediatric patients, where it could help prevent amblyopia and strabismus. These encouraging preliminary results warrant further validation through larger case series and long-term follow-up, positioning this technique as a significant advancement and a potentially more accessible and less invasive solution.

## Figures and Tables

**Movie 1:** 

**Table 1:** Summary of surgical steps and key comments.

Pupiloplastia Retroiridiana Fáquica: Paso a paso.		
Step	Surgery	Key Comments
**1. Antiseptic Preparation**	Application of 5% povidone-iodine solution to the face, ocular surface, and conjunctival fornix.	Avoid in patients with iodine allergy.
**2. Topical Anesthesia**	Instillation of 0.5% proparacaine hydrochloride drops.	In order to preserve natural pupillary behavior during positioning of the neopupil. The procedure was performed under intravenous neuroleptanesthesia.
**3. Limbal Incisions**	Creation of two 0.8 mm limbal incisions, temporally and nasally, using a 15° blade.	Minimal incision size is recommended to prevent iris prolapse and anterior chamber collapse.
**4. Endothelial Protection**	Injection of dispersive viscoelastic, occupying one-third of the anterior chamber, anterior to the iris.	2% hydroxypropyl methylcellulose was used to protect corneal endothelial cells during intraocular manipulation.
**5. Access to the Retroiridian Space**	Creation of a small peripheral iridectomy using Westcott scissors and Castroviejo forceps.	This provides access to the retroiridian space. May alternatively be performed preoperatively with Nd:YAG laser.
**6. Molding of the Retroiridian Space**	Injection of cohesive viscoelastic through the iridectomy to form a superior convex dome.	3% sodium hyaluronate was used to expand and stabilize the retroiridian space, displacing the iris away from the crystalline lens.
**7. Anterior Chamber Entry**	Insertion of a continuous irrigation handpiece via the non-dominant hand.	Irrigation was kept off until the vitrectome was correctly threaded through the iridectomy.
**8. Phakic Retroiridian Pupilloplasty**	Insertion of a 20G anterior vitrectome through the iridectomy using the dominant hand. Parameters: 1000 cpm, 20 cc/min aspiration, 250 mmHg vacuum.	Create the neopupil in the retroiridian space. Angled upward so that its silhouette was visible through the iris, avoiding contact with the crystalline lens.
**9. Viscoelastic Aspiration**	Aspiration of viscoelastic from the anterior chamber.	Performed anterior to the iris, avoiding contact with the crystalline lens.
**10. Incision Closure**	Hydration of incisions and iris entrapment at the iridectomy site.	Iris entrapment into the corneal stroma at one-third depth avoids the need for sutures and reduces the size of the iridectomy.

**Table 2:** Key advantages of the phakic retroiridian pupilloplasty technique.

Some Techniques for Pupillary Reconstruction: From Anterior Chamber Approaches to Retroiridian Pupilloplasty			
Technique	Entry Point	Description	Limitations/Risks
**Nd:YAG Laser**	Anterior chamber approach	Photodisruption of pupillary membranes	Variable outcomes; risk of damage to adjacent tissues: hyphema, cataract formation, iritis, and pigment dispersion
**Vitrectome, Microforceps, Microsurgical Scissors**	Anterior chamber approach	Removal of iris tissue often associated with membranes	Complex maneuvers; risk of damage to adjacent tissues; limited visibility due to bleeding and instruments
**Endothermal Pupilloplasty**	Anterior chamber approach	Pupil displacement by thermal contraction of iris using bipolar cautery (± Siepser sutures)	Risk of adjacent tissue damage due to high temperatures such as hyphema, cataract, iritis, and iris depigmentation; ineffective in acoria; limited visibility due to bubbles
**Phakic Retroiridian Pupilloplasty**	Retroiridian space approach	Creation of a neopupil, away from the crystalline lens	We approach represents the first implementation of a retroiridian access in phakic eyes. New technique: does not require advanced prior experience, had no complications reported, and provided good surgical visibility

**Table 3:** Advantages of phakic retroiridian pupilloplasty.

Comparison of Phakic Retroiridian Pupilloplasty *vs.* Commonly Used Techniques		
Characteristic	Phakic Retroiridian Pupilloplasty	Common Pupilloplasty Techniques
**Lens Preservation (Phakic Eyes)**	Performed with the native, healthy crystalline lens in place.	Often require lens extraction due to technical limitations.
**Surgical Invasiveness**	Minimally invasive; a neopupil is created behind the iris using a vitrector through a small peripheral iridectomy.	More invasive; involve iris suturing, increased manipulation, and sometimes thermal energy.
**Dissection Plane**	Peripheral; avoids contact with the crystalline lens.	Central; in direct contact with the lens.
**Complexity and Learning Curve**	Simple, reproducible, and associated with a short learning curve.	Some techniques are highly complex, requiring advanced skills and prolonged training.
**Risk of Complications**	In our series (2022–present), no reports of intraoperative bleeding, iris atrophy, or cataract induction.	Higher risk of bleeding, significant inflammation, synechiae, iris atrophy, or cataract formation.
**Required Instrumentation**	Uses standard ophthalmic surgical instruments (viscoelastic, vitrectome, scissors, forceps); readily available.	Some techniques require specialized instruments and sutures, limiting accessibility.
**Functional and Aesthetic Restoration**	Predictable long-term functional and cosmetic outcomes.	Long-term outcomes may be less predictable.

## Data Availability

The datasets during and/or analyzed during the current study available from the corresponding author on reasonable request.
